# Subgroups of High-Cost Patients and Their Preventable Inpatient Cost in Rural China

**DOI:** 10.34172/ijhpm.2024.8151

**Published:** 2024-03-09

**Authors:** Shan Lu, Yan Zhang, Ting Ye, Dionne S. Kringos

**Affiliations:** ^1^School of Medicine and Health Management, Tongji Medical College, Huazhong University of Science and Technology, Wuhan, China.; ^2^Research Centre for Rural Health Service, Key Research Institute of Humanities & Social Sciences of Hubei Provincial Department of Education, Wuhan, China.; ^3^Amsterdam Public Health Research Institute, Department of Public and Occupational Health, University of Amsterdam, Amsterdam UMC, Amsterdam, The Netherlands.

**Keywords:** High-Cost Patients, Patient Segmentation, Potentially Preventable Hospitalization, Preventable Inpatient Cost, Rural China

## Abstract

**Background:** High-cost patients account for most healthcare costs and are highly heterogeneous. This study aims to classify high-cost patients into clinically homogeneous subgroups, describe healthcare utilization patterns of subgroups, and identify subgroups with relatively high preventable inpatient cost (PIC) in rural China.

**Methods:** A population-based retrospective study was performed using claims data in Xi county, Henan province. 32 108 high-cost patients, representing the top 10% of individuals with the highest total spending, were identified. A density-based clustering algorithm combined with expert opinions were used to group high-cost patients. Healthcare utilization (including admissions, length of stay, and outpatient visits) and spending characteristics (including total spending, and the proportion of PIC, inpatient and out-of-pocket spending on total spending) were described among subgroups. PIC was calculated based on potentially preventable hospitalizations (PPHs) which were identified according to the Agency for Healthcare Research and Quality Prevention Quality Indicators algorithm.

**Results:** High-cost patients were more likely to be older (Mean=51.87, SD=22.28), male (49.03%) and from poverty-stricken families (37.67%) than non-high-cost patients, with 2.49 (SD=2.47) admissions and 3.25 (SD=4.52) outpatient visits annually. Fourteen subgroups of high-cost patients were identified: chronic disease, non-trauma diseases which need surgery, female disease, cancer, eye disease, respiratory infection/inflammation, skin disease, fracture, liver disease, vertigo syndrome and cerebral infarction, mental disease, arthritis, renal failure, and other neurological disorders. The annual admissions ranged from 1.83 (SD=1.23, fracture) to 12.21 (SD=9.26, renal failure), and the average length of stay ranged from 6.61 (SD=10.00, eye disease) to 32.11 (SD=28.78, mental disease) days among subgroups. The chronic disease subgroup showed the largest proportion of PIC on total spending (10.57%).

**Conclusion:** High-cost patients were classified into 14 clinically distinct subgroups which had different healthcare utilization and spending characteristics. Different targeted strategies may be needed for subgroups to reduce preventable hospitalizations. Priority should be given to high-cost patients with chronic diseases.

## Background

**Key Messages**

**Implications for policy makers**

- This study classifies high-cost patients into homogeneous subgroups and identifies subgroups with relatively high preventable costs, improving the understanding of the high-cost population in rural China, thereby facilitating a more meaningful discussion about reducing healthcare costs and enhancing health outcomes.
- Priority could be given to developing strategies for the chronic disease group of which preventable inpatient costs (PICs) accounted for more than 10% of total spending.
- The high cost for non-trauma disease which need surgery was avoidable if primary care could be well-utilized, and more effective actions to reduce reproductive system diseases for women are needed in rural China.
- Dialysis services can be transferred to outpatient settings and even primary care facilities to increase accessibility and reduce the economic burden for high-cost patients with renal failure.

**Implications for the public**

Understanding high-cost patients better is critical to improving health outcomes, reducing healthcare costs and increasing efficiency. Studies showed that high-cost patients were heterogeneous. However, knowledge from low- and middle-income countries, including China, of segmenting high-cost patients into operationally significant subgroups is lacking. This study indicates that high-cost patients from rural China can be classified into 14 clinically distinct subgroups with a significant difference in the number of admissions (ranging from 1.83 to 12.21), average length of stay (ranging from 6.11 to 32.11 days) and proportion of preventable inpatient cost (PIC) on total healthcare spending (ranging from 1.21% to 10.57%) in 2019. The high costs for some subgroups of patients were avoidable if primary care could be trusted and utilized more.

In the past decades, health spending kept rising in most countries with population ageing, economic progress, medical technological advancements and epidemiological transitions.^1^ The COVID-19 pandemic has added to the financial pressure because responding to the pandemic has been and continues to be tremendously costly, and the economic consequences of the health crisis are leading to long-standing reductions in economic development in some countries.^1-3^ Despite uncertainty, spending on health is expected to continue to grow in the future, albeit at a slower pace than anticipated before the pandemic.^1^ China was no exception. The average annual growth rate of total health expenditure was higher than that of gross domestic product from 2011 to 2019, and the COVID-19 pandemic has only increased the rate. High-cost patients, the costliest small proportion of patients who account for a disproportionate amount of total health spending, are attracting the interests of governments, health insurers, providers and healthcare researchers in recent years.^4,5^ Understanding this small percentage of the patient cohort better might be critical to improving health outcomes, reducing healthcare costs and increasing efficiency, hence contributing to the financial sustainability of the health system.^6^

 Empirical evidence from different countries demonstrated that high-cost patients are characterized by repeated hospitalizations, and inpatient costs account for a large proportion of their total healthcare spending.^7-9^ According to our previous study on the rural population, the occurrence of potentially preventable hospitalization (PPH) among high-cost patients was sizable (22%), indicating that 22 preventable hospitalizations occurred per 100 high-cost persons.^10^ Moreover, the preventable inpatient cost (PIC) of high-cost patients amounted to the majority (around 70%) of total preventable spending of overall patients as previous studies reported.^6,10-12^ Although high-cost patients only occupy a small proportion of the population, they are highly heterogeneous, which has substantial variations in demographics, functional status and disease burden.^7,13,14^ Not all hospitalizations are potentially preventable among high-cost patients. For patients with severe trauma who need acute surgery treatment or patients with cancer who need expensive radiotherapy and chemotherapy, there may be limited opportunities to reduce spending.^14^ Previous studies indicated that the prerequisite to reduce spending for high-cost patients is to identify their difference and then implement targeted interventions for subgroups.^7,15^ Therefore, classifying high-cost patients into homogeneous groups and paying more attention to those with higher preventable spending are necessary.

 Most existing studies on segmenting high-cost patients were based on expert opinions, indicating that there may be opportunities to supplement these approaches and strengthen the evidence base by identifying subgroups using data-driven methods. To our knowledge, only two studies on Medicare Advantage beneficiaries and Medicare Fee-for-service beneficiaries identified subgroups of high-cost patients exclusively based on the analysis of variation within patient data from the United States.^14,16^ One of them focused on clinical distinction among high-cost patients and another was concerned with patterns of high-cost healthcare utilization. Knowledge, from low- and middle-income countries, including China, of segmenting high-cost patients into operationally significant subgroups is lacking. Patient taxonomy from high-income countries may not apply to high-cost populations in China. Our previous study showed that high-cost patients from rural China presented remarkable clinical variations. Therefore, this study aims to classify high-cost patients into homogeneous subgroups according to demographic and clinical characteristics, describe healthcare utilization patterns of subgroups of high-cost patients and identify subgroups of high-cost cohorts with relatively high PIC in rural China.

 We address the following research questions:

1. What are the socio-demographic, clinical and healthcare utilization characteristics of high-cost and non-high-cost patients from rural China?
2. Can high-cost patients be classified into homogeneous subgroups according to demographic and clinical characteristics?
3. What are the utilization patterns of subgroups of high-cost patients?
4. Which subgroups of high-cost patients have relatively high PIC?

## Methods

###  Study Design and Sample

 This population-based retrospective study was performed in Xi county, Henan province. Xi is a rural area located in central China and covers an area of 1892 km^2^. Xi has 0.42 million rural residents and a gross regional product per capita of US$ 5078.3 in 2019 (exchange rate in 2019: CNY 6.90 to US$ 1.00), which is about half of the gross domestic product per capita of China (US$ 10274.2). More than 95% of the rural residents were covered by the Basic Medical Insurance for Urban and Rural Residents (BMIUR), which offered reimbursement for outpatient and inpatient services in healthcare facilities at different levels. The hierarchical healthcare system in rural China mainly consists of three levels of healthcare facilities: village clinics, township health centres and county hospitals. Xi has 338 village clinics, 22 township health centres and 4 county hospitals (including two general hospitals, a maternal and child health hospital and a traditional Chinese medicine hospital). Village clinics and township health centres provide primary care. The latter also provides inpatient services, just as county hospitals. The BMIUR database records healthcare utilization and health expenditure, encompassing total health expenditure and out-of-pocket health expenditure, for every rural resident under BMIUR coverage.

 According to the 2019 BMIUR database, 321 082 rural patients who utilized outpatient or inpatient services were involved in this study. Patients in the highest 10% of total individual spending (including inpatient and outpatient spending) were defined as high-cost patients. The top 10% of patients were identified in accordance with previous studies on preventable spending of high-cost patients.^6,11,17^ The study population consisted of 32 108 high-cost patients, who accounted for 73.88% of total spending.

###  Data

 We extracted data from the 2019 BMIUR database (with outpatient and inpatient data) for Xi county, Henan province. A full description of study variables is provided in Supplementary file 1. Variables included in this study can be grouped into the following categories: socio-demographics including demographics and economic status, clinical characteristics including active diagnosis, chronic conditions and departments, and outcomes including utilization and spending. Demographic variables included age and gender. Economic status was represented by family income (ie, from a poverty-stricken family or not). Patients were classified into poverty-stricken family if they lived below the national poverty line. We classified patients into poverty-stricken family or non-poverty-stricken family according to the 2019 BMIUR database. Active diagnosis was the principal diagnosis for each outpatient visit or admission in 2019. Principal diagnoses were categorized according to the China Healthcare Security Diagnosis Related Groups (CHS-DRG) for each patient. Based on the similarity of clinical process and resource consumption, CHS-DRG categorizes the 10th revision of the International Classification of Diseases 10th Revision (ICD-10) codes into 187 adjacent diagnosis-related groups (ADRGs), such as open brain injury and hypertension. Therefore, we finally got 187 ADRGs for active diagnosis. Chronic conditions were identified using the 2017–2019 BMIUR databases and categorized into 31 groups according to the Elixhauser classification. Departments represent where the patients were hospitalized in 2019 and include the internal medicine, surgery, orthopaedics, gynaecology, obstetrics, paediatrics, oncology, ophthalmology and ENT (ear, nose, and throat) and stomatology, and rehabilitation departments. Utilization and spending variables were calculated for each patient using the 2019 BMIUR databases. The list of the 187 ADRGs and the 31 groups of chronic conditions is shown in Supplementary file 2.

###  Identify Subgroups 

 Clustering is an unsupervised machine learning technique that groups observations (eg, patients) according to similarities among measured characteristics. Clustering algorithms iteratively group observations into clusters until they find the allocation that maximizes both intra-group similarity and inter-group differences. Density-based clustering with the ordering points to identify the clustering structure (OPTICS) algorithm was used to classify high-cost patients.^14^ Variables used for clustering included demographics, active diagnosis, chronic conditions, and departments (See Supplementary file 1 for specific variables). In total, 229 variables were involved in clustering. Utilization and spending variables were not used for clustering, allowing for a comparison of healthcare utilization and spending patterns across clusters.

 To perform cluster analysis, we began by analytically reducing the number of variables in the dataset. We removed variables with extremely low variance following the criteria from a previous related study.^18^ To reduce outliers, we removed ADRGs with less than 1% prevalence and comorbidities with less than 0.1% prevalence. A total of 91 binary variables were retained, and a comprehensive list is available in Supplementary file 2. Principal component analysis was used to further reduce the number of variables, and 58 components which explained 80% of the variability in the original data were chosen. Then,we used a validated, non-linear dimension reduction algorithm called t-distributed stochastic neighbour embedding (t-SNE) to create a low-dimension representation of the dataset.^18^ The t-SNE method takes as input a high-dimensional data set and maps each observation to a lower-dimensional space. We ran a specific implementation of t-SNE known as the Barnes–Hut algorithm and mapped it to a two-dimensional space to facilitate visualization.^18^ Finally,we employed clustering algorithms on the low-dimensional dataset, specifically with two variables. We followed a standardized approach described by Yan and colleagues^18^ for tuning model parameters. We restricted algorithm solutions to those that yielded at least five clusters. We restricted the minimum number of patients per cluster to at least 1% of the high-cost patients to ensure that the clusters were operationally meaningful.

 Our rationale for choosing the OPTICS algorithm was that Yan and colleagues’ similar study^18^ showed that OPTICS algorithm outperformed connectivity-based and centroid-based clustering algorithms. To verify the performance of OPTICS with our data, other two clustering algorithms were also used: connectivity-based clustering using agglomerative hierarchical clustering and centroid-based clustering with the k-medoids algorithm.^18^ We evaluated the performance of the three algorithms by (*a*) performing a visual examination of the cluster assignments using the two-dimensional representation of the data set generated by the t-SNE algorithm and (*b*) adopting a set of ridge regression models to better understand the relationship between cluster assignment and clinical variables (See Yan and colleagues’ research^18^ for details of evaluating the algorithm performance). Consistent with Yan and colleagues’ research,^18^ the OPTICS algorithm showed the best performance in this study. The results of algorithm performance evaluation are presented in Supplementary file 3. As data-driven methods may not always yield perfect results, we enhanced intra-group similarity and inter-group differences by consulting with three physicians to refine the clustering outcomes. We finally identified the subgroups of high-cost patients based on the results of clustering and expert opinions.

###  Preventable Inpatient Cost

 PIC in 2019 encompassed the total expenditure, inclusive of BMIUR-reimbursed and out-of-pocket spending, for PPHs. The ICD-10 codes for principal diagnosis of hospitalizations were extracted from the BMIUR database. PPHs were identified according to the Agency for Healthcare Research and Quality Prevention Quality Indicators algorithm, which defines PPHs as those related to conditions, such as heart failure, diabetes, hypertension, and asthma, for which good outpatient care can likely prevent the need for hospitalization.^19^ The tool was validated and used in prior work on populations in China.^10,20^ The list of ICD-10 codes used to identify PPHs is shown in Supplementary file 4.

###  Cluster and Subgroup Analysis

 To delineate the characteristics of the resulting clusters from the algorithm, we initially computed means for both the overall high-cost population involved in the clustering analysis and the cluster-specific means for each variable used in the clustering process.^14^ We then calculated standardized ratios of cluster means to population means, such that larger numbers represented variables for which the cluster deviated most from the broader high-cost population.^14^ We assigned a descriptive label to each cluster based on the variables with the highest standardized ratios as well as variables for which the ratios varied most among clusters. Given the numerous variables, we chose to present the three to five variables with the largest standardized ratios (labelled as distinguishing factors) for each cluster.^14^ We compared healthcare utilization (including admissions, length of stay and outpatient visits) and spending (including total spending, and the proportion of PIC, inpatient and out-of-pocket spending on total spending) across subgroups of high-cost patients. R 4.1.1 was used for clustering. The Rtsne package version 0.16 was used for t-SNE and the dbscan package version 1.1–11 was used for OPTICS algorithm. The cluster package version 2.1.4 was used for agglomerative hierarchical clustering with Ward’s criterion and k-medoids algorithm. Stata 15.1 was used for other analyses.

## Results

###  Study Sample

Table 1 presents the socio-demographic, clinical, healthcare utilization and spending characteristics of the study sample. The average age of high-cost patients (51.87 years) was around 10 years older than the non-high-cost patients (41.44 years). Females accounted for 50.97% of the high-cost group, which is slightly lower than that of the non-high-cost group. The proportion of patients from poverty-stricken families among high-cost patients (37.67%) is higher than that of non-high-cost patients (16.26%). On average, high-cost patients annually experienced 2.47 admissions and 3.25 outpatient visits, which were both more than that of the non-high-cost patients (0.37 admissions and 2.80 visits). The average length of stay of high-cost patients (10.80 days) was longer than that of the non-high-cost patients (1.59 days). The top 10 chronic conditions of high-cost patients were chronic pulmonary disease, hypertension (uncomplicated), diabetes (uncomplicated), solid tumours without metastasis, congestive heart failure, diabetes (complicated), psychoses, liver disease, peptic ulcer disease excluding bleeding and renal failure. Among their top 10 ADRGs, high-cost patients had a remarkably higher burden of respiratory infection/inflammation, coronary atherosclerosis/thrombus/occlusion, cerebral ischemic disease, neoplasms and hypertension than non-high-cost patients. The average total spending (US$ 3898.06) and PIC (US$ 235.27) of high-cost patients were much higher than that of non-high-cost patients.

**Table 1:** Study Sample

	**Overall** ** (n = 321** **082)**	**High-Cost Patients** ** (n = 32** **108)**	**Non-high-Cost Patients** **(n = 288** **974)**
Age (mean, SD)	42.48 (22.60)	51.87 (22.28)	41.44 (22.39)
Gender, No. (%)			
Male	155 550 (48.45)	15 742 (49.03)	139 808 (48.38)
Family income, No. (%)			
Poverty-stricken	59 078 (18.40)	12 095 (37.67)	46 983 (16.26)
Admissions (mean, SD)	0.58 (1.20)	2.49 (2.47)	0.37 (0.68)
Admissions within county (mean, SD)	0.50 (1.08)	1.83 (2.36)	0.35 (0.67)
Admissions outside county (mean, SD)	0.08 (0.47)	0.67 (1.29)	0.02 (0.13)
Admissions, No. (%)			
0	207 593 (64.65)	402 (1.25)	207 191 (71.7)
1	76 792 (23.92)	13 386 (41.69)	63 406 (21.94)
2	21 834 (6.80)	7853 (24.46)	13 981 (4.84)
≥3	14 863 (4.63)	10 467 (32.60)	4396 (1.52)
Average LOS (mean, SD)	2.51 (6.08)	10.80 (12.85)	1.59 (3.78)
Outpatient visits (mean, SD)	2.84 (3.2)	3.25 (4.52)	2.80 (3.02)
Visits within county (mean, SD)	2.84 (3.2)	3.22 (4.48)	2.80 (3.02)
Visits outside county (mean, SD)	0.003 (0.16)	0.03 (0.5)	0.0002 (0.02)
Top 10 chronic conditions of high-cost patients, No. (%)			
Chronic pulmonary disease	65 050 (20.26)	6548 (20.39)	58 355 (20.19)
Hypertension, uncomplicated	44 134 (13.75)	5895 (18.36)	38 222 (13.23)
Diabetes, uncomplicated	18 418 (5.74)	3210 (10.00)	15 190 (5.26)
Solid tumour without metastasis	4078 (1.27)	2926 (9.11)	1149 (0.40)
Congestive heart failure	3868 (1.20)	2417 (7.53)	1448 (0.50)
Diabetes, complicated	2800 (0.87)	838 (2.61)	1962 (0.68)
Psychoses	2266 (0.71)	821 (2.56)	1434 (0.50)
Liver disease	1769 (0.55)	627 (1.95)	1141 (0.39)
Peptic ulcer disease excluding bleeding	3390 (1.06)	621 (1.93)	2768 (0.96)
Renal failure	510 (0.16)	456 (1.42)	53 (0.02)
Top 10 ADRGs of high-cost patients			
Upper respiratory disease^a^	95 986 (29.89)	6054 (18.86)	89 932 (31.12)
Other neurological disorders^b^	46 625 (14.52)	5297 (16.50)	41 328 (14.30)
Respiratory infection/inflammation^c^	23 669 (7.37)	4159 (12.95)	19 510 (6.75)
Obstruction of digestive tract or abdominal pain	34 962 (10.89)	3897 (12.14)	31 065 (10.75)
Esophagitis, gastroenteritis	35 650 (11.10)	3766 (11.73)	31 884 (11.03)
Neck and back disease^d^	26 810 (8.35)	3390 (10.56)	23 420 (8.10)
Coronary atherosclerosis/thrombus/occlusion^e^	9830 (3.06)	3344 (10.41)	6486 (2.24)
Cerebral ischemic disease^f^	11 544 (3.60)	3252 (10.13)	8292 (2.87)
Neoplasms	3600 (1.12)	3138 (9.77)	462 (0.16)
Hypertension	24 994 (7.78)	3075 (9.58)	3075 (1.06)
Total spending (mean, SD), US$	527.63 (2046.28)	3898.06 (5363.85)	153.14 (230.91)
PIC (mean, SD), US$	38.11 (289.84)	235.27 (855.05)	16.20 (85.50)

Abbreviations: SD, standard deviation; PIC, preventable inpatient cost; ADRGs, adjacent diagnosis-related groups; LOS, length of stay. ^a^Upper respiratory disease (J39.900) belongs to ADRG “other head, neck, ear, nose, pharyngeal, mouth diseases.” More than 95% of the principal diagnosis for this ADRG were upper respiratory disease (and were from outpatient visits), so we present “upper respiratory disease” instead of “other head, neck, ear, nose, pharyngeal, mouth diseases” to clarify the disease. ^b^Other neurological disorders were represented by vertigo and dizziness (R42.x00), sequelae of cerebral infarction (I69.300) and cerebrovascular disease (I67.900). ^c^Respiratory infection/inflammation was represented by bronchopneumonia (J18.000), Community-acquired pneumonia (J15.902). ^d^Neck and back disease was represented by lumbar disc herniation (M51.202), lumbago (M54.502) and cervical disc herniation (M50.201). ^e^Coronary atherosclerosis/thrombus/occlusion was represented by coronary atherosclerotic heart disease (I25.103). ^f^Cerebral ischaemic disease was represented by cerebral infarction (I63.900).

###  Subgroups of High-Cost Patients

 We disallowed duplicate samples, defined as samples sharing identical values for each variable used in clustering; therefore, 18 434 high-cost patients were included in cluster analysis after removing duplicates. Clustering identified 31 clusters of high-cost patients. The number of patients in each cluster ranged from 182 (0.99%) to 5489 (29.78%), and 2831 patients (15.36%) were not assigned to any cluster. To reduce the number of clusters and thus increase operational meaning, clusters with clinical similarities were merged into larger subgroups according to the suggestions of three physicians. Finally, 31 clusters were merged into 14 subgroups. The clusters from cluster analysis and subgroups from physicians’ opinions are shown in Table 2. The visual representation of patient clusters was shown in Supplementary file 3.

**Table 2:** Description of High-Cost Clusters and Subgroups

**Subgroups**	**Clusters**	**Distinguishing Factor**	**Category**
Chronic disease 42.49%	Mixed chronic disease 29.78%	Diabetes, complicated	Comorbidity
		Angina pectoris	ADRG
		Diabetes	ADRG
		Peptic ulcer disease excluding bleeding	Comorbidity
		Cerebral ischemic disease	ADRG
	Heart failure and COPD 4.43%	Heart failure, shock	ADRG
		Congestive heart failure	Comorbidity
		Chronic obstructive airway disease	ADRG
	Hyperlipidemia and hypertension 2.00%	Hyperlipidemia	ADRG
		Hypertension	ADRG
		Sequela of cerebrovascular disease	ADRG
	Cerebrovascular disease with headache 1.72%	Headache	ADRG
		Intracranial haemorrhage	ADRG
		Cerebral ischemic disease	ADRG
	Rehabilitation of cerebrovascular diseases 1.70%	Intracranial haemorrhage	ADRG
		Other rehabilitation treatment	ADRG
		Rehabilitation	Department
	Circulatory system disorders with chest pain 1.63%	Chest pain	ADRG
		Other circulatory system disorders	ADRG
		Coronary atherosclerosis/thrombus/occlusion	ADRG
	Cardiac arrhythmias and CHD 1.23%	Cardiac arrhythmias	Comorbidity
		Arrhythmia and conduction disorder	ADRG
		Coronary atherosclerosis/thrombus/occlusion	ADRG
Non-trauma diseases which need surgery 13.47%	Urinary calculi 3.56%	Urinary calculi, obstruction, and urethral stricture	ADRG
		Other diseases of kidney and urinary system	ADRG
		Surgery	Department
	Digestive system diseases which need surgery (Such as appendicitis, haemorrhoids, polyps, and hernias) 2.53%	Other digestive system diagnosis	ADRG
		Surgery	Department
		Obstruction of digestive tract or abdominal pain	ADRG
	Gallstone and cholecystitis 1.94%	Other diseases of biliary tract	ADRG
		Acute biliary tract disease	ADRG
		Surgery	Department
	Disease of male reproductive system (eg, prostate hyperplasia) 1.70%	Other male reproductive system disorders	ADRG
		Renal and urinary tract infection	ADRG
		Surgery	Department
	Venous diseases which need surgery (varicosity) 1.39%	Venous disease	ADRG
		Surgery	Department
		Major skin disorders	ADRG
	Thyroid disorders 1.36%	Endocrine disorders	ADRG
		Oncology	Department
		Surgery	Department
	Non-malignant hyperplasia of head, neck, ear, nose, pharynx, or mouth 0.99%	Head, neck, ear, nose, pharynx and mouth are non-malignant proliferative	ADRG
		Ophthalmology, ENT, stomatology	Department
		Oral and dental related diseases	ADRG
Female disease 4.54%	Disease of female reproductive system (eg, myoma of uterus) 2.98%	Gynaecology	Department
		Female reproductive infection	ADRG
		Other diseases of female reproductive system	ADRG
	Benign breast lesions (breast lumps/abscesses) 1.56%	Benign breast lesions	ADRG
		Gynaecology	Department
		Female reproductive infection	ADRG
Cancer 4.18%	Digestive system tumour 2.50%	Digestive system malignant tumour	ADRG
		Solid tumour without metastasis	Comorbidity
		Radiotherapy for malignant proliferative diseases	ADRG
	Respiratory system tumour 1.68%	Respiratory system tumour	ADRG
		Solid tumour without metastasis	Comorbidity
		Radiotherapy for malignant proliferative diseases	ADRG
Eye disease 3.80%	Cataract 2.02%	Cataract of various types	ADRG
		Ophthalmology, ENT, stomatology	Department
		Diabetes, complicated	Comorbidity
	Other eye diseases (such as retinal disorders) 1.77%	Other eye diseases	ADRG
		Ophthalmology, ENT, stomatology	Department
		Cataract of various types	ADRG
Respiratory infection/inflammation 3.58%	Respiratory infection/inflammation with fever 2.08%	Fever with unknown cause	ADRG
		Respiratory infection/inflammation	ADRG
		Upper respiratory tract infection and tympanitis	ADRG
	Child respiratory infection/inflammation (pneumonia) 1.50%	Paediatrics	Department
		Upper respiratory tract infection and tympanitis	ADRG
		Respiratory infection/inflammation	ADRG
Skin disease 2.57%	Inflammatory dermatosis 1.37%	Inflammatory dermatosis	ADRG
		Asthma and asthmatic bronchitis	ADRG
		Other digestive system diagnosis	ADRG
	Major skin disorders (such as herpes zoster) 1.20%	Major skin disorders	ADRG
		Other bones, muscles, tendons, connective tissue	ADRG
		Ophthalmology, ENT, stomatology	Department
Fracture 1.99%	Fracture 1.99%	Injury except forearm, wrist, hand and foot	ADRG
		Orthopedics	Department
		Other bones, muscles, tendons, connective tissue	ADRG
Liver disease 1.89%	Liver disease 1.89%	Liver disease	Comorbidity
		Solid tumour without metastasis	Comorbidity
		Radiotherapy for malignant proliferative diseases	ADRG
Vertigo syndrome, fever, and cerebral infarction 1.42%	Vertigo syndrome, fever, and cerebral infarction 1.42%	Imbalance and hearing disorders	ADRG
		Fever with unknown cause	ADRG
		Cerebral ischemic disease	ADRG
Mental disease 1.31%	Mental disease 1.31%	Schizophrenia	ADRG
		Psychoses	Comorbidity
		Depression	Comorbidity
Arthritis 1.25%	Arthritis 1.25%	Rheumatoid arthritis/collagen vascular diseases	Comorbidity
		Osteopathy and other joint diseases	ADRG
		Orthopedics	Department
Renal failure 1.21%	Renal failure 1.21%	Renal insufficiency	ADRG
		Renal failure	Comorbidity
		Renal and urinary tract infection	
Other neurological disorders 0.99%	Other neurological disorders 0.99%	Other neurological disorders	Comorbidity
		Other rehabilitation treatment	ADRG
		Other neurological disorders	ADRG

Abbreviations: ADRG, adjacent diagnosis-related group; COPD, chronic obstructive pulmonary disease; CHD, coronary heart disease; ENT, ear, nose, and throat.

###  Utilization and Spending for High-Cost Patient Subgroups

 We compared the healthcare utilization and spending for both high-cost patient clusters (ie, 31 clusters) and subgroups (ie, 14 subgroups). The healthcare utilization and spending for the 14 high-cost patient subgroups and 31 clusters are shown in Figure and Supplementary file 5, respectively. Figure shows that annual admissions ranged from 1.83 to 12.21 among high-cost patient subgroups. Patients from the fracture subgroup had the smallest admissions (1.83) on average, while the renal failure disease subgroups presented the largest number of admissions (12.21). The eye disease subgroup showed the shortest length of stay (6.61 days), and patients from the mental disease subgroup experienced 32.11 days on average for each admission. The average outpatient visits ranged from 4.09 to 7.83 among high-cost patient subgroups. The skin disease subgroup and vertigo syndrome and fever and cerebral infarction subgroup showed more than 7 outpatient visits in 2019.

**Figure:**
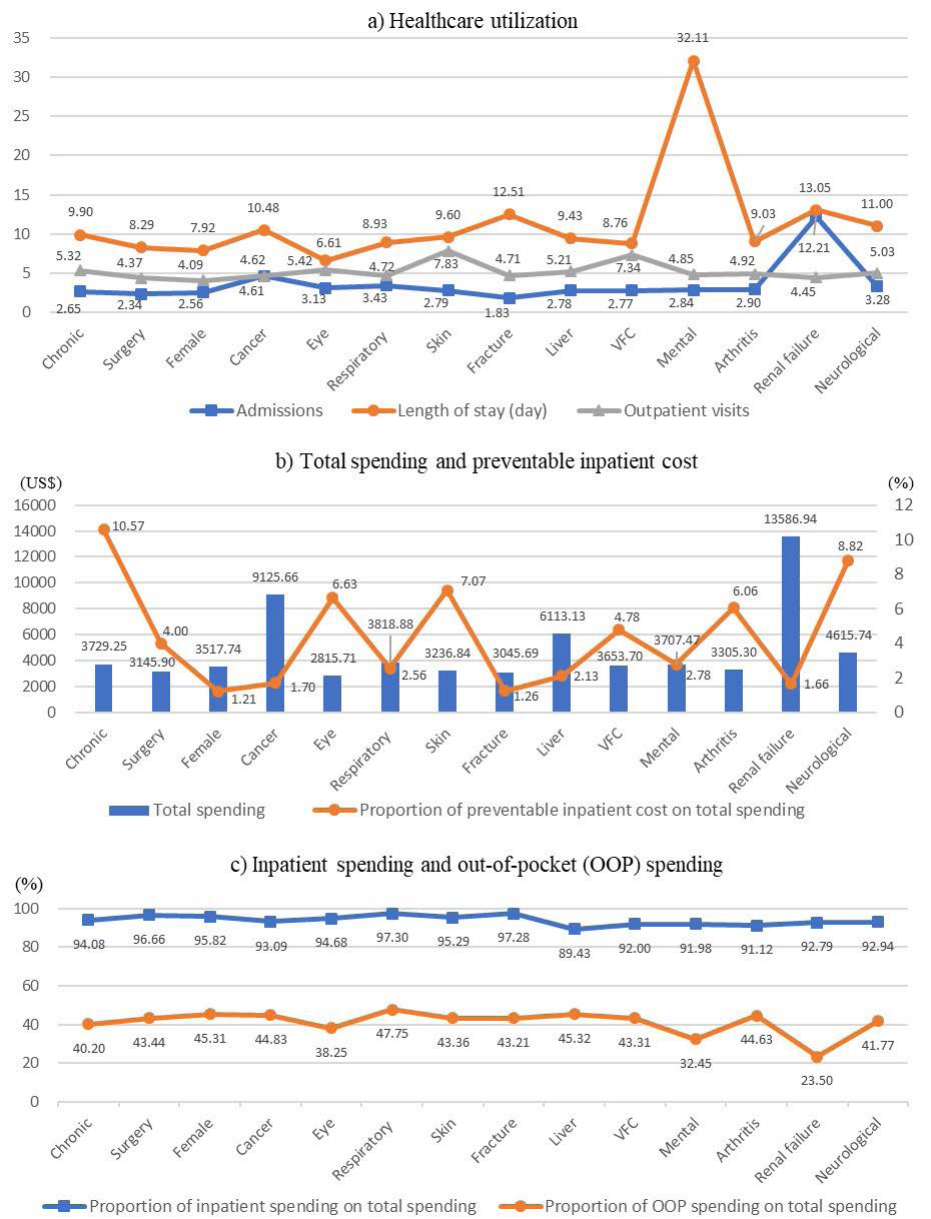
Utilization and Spending for High-Cost Patient Subgroups.Abbreviations: VFC, Vertigo syndrome & fever & cerebral infarction; OOP, out-of-pocket.

 The average total spending for the 14 high-cost patient subgroups ranged from US$ 2815.71 to US$ 13 586.94. The three subgroups with the highest total spending were the renal failure subgroup (US$ 13 586.94), cancer subgroup (US$ 9125.66) and liver disease subgroup (US$ 6113.13). The proportion of PICs on total spending ranged from 1.21% to 10.57% among the 14 subgroups. The proportions of PICs on total spending for the chronic disease subgroup were more than 10% (10.57%). By contrast, the proportions of PIC on total spending for the female disease subgroup (1.21%), cancer subgroup (1.70%), fracture subgroup (1.26%) and renal failure subgroup (1.66%) were low. Inpatient spending accounted for more than 90% of total spending for almost all of the 14 subgroups, except for the liver disease subgroup (89.43%). Out-of-pocket spending amounted to 23.50%–47.75% of total spending among the high-cost patient subgroups. The renal disease subgroup showed the smallest proportion of out-of-pocket spending on total spending, while 11 out of 14 subgroups presented more than 40% of out-of-pocket spending on total spending.

## Discussion

 This study aimed to classify high-cost patients into homogeneous subgroups, describe healthcare utilization and spending of subgroups and identify subgroups of high-cost patients with relatively high PIC in rural China. High-cost patients (average age of 51 years) were ten years older than non-high-cost patients, with annually 2.47 admissions and 3.25 outpatient visits. The top 10 chronic conditions of high-cost patients were chronic pulmonary disease, hypertension (uncomplicated), diabetes (uncomplicated), solid tumour without metastasis, congestive heart failure, diabetes (complicated), psychoses, liver disease, peptic ulcer disease excluding bleeding and renal failure. A total of 31 clusters of high-cost patients were identified using cluster analysis, and then 31 clusters were merged into 14 larger subgroups according to experts’ opinions to increase operational meaning. The 14 subgroups of high-cost patients presented significant differences in the number of admissions (ranging from 1.83 to 12.21) and average length of stay (ranging from 6.11 to 32.11 days) in 2019. The chronic disease subgroup showed the largest proportion of PICs on total spending, which was 10.57%.

 High-cost patients were older than non-high-cost patients, as proven by studies from different countries.^10,12,21,22^ However, studies showed inconsistent results for gender. This study showed slightly fewer female patients in the high-cost group, which is even contrary to the result of our previous study from a different city in rural China.^10^ The proportion of patients from poverty-stricken families among the high-cost group was around twice that among the non-high-cost group, which was consistent with our previous study.^10^ This may result from the lower willingness to seek healthcare and the worse health status of poverty-stricken patients,^23,24^ and the healthcare spending tended to be high when they were badly sick and sought healthcare. Socioeconomic status is a predictor of high costs, as a Canadian study showed that high costs were most strongly related to food insecurity, lower income, non-homeownership and living in a highly deprived neighbourhood.^25^ Around 33% of high-cost patients experienced three or more admissions, and 42% of high-cost patients had only one admission in 2019, indicating that both repeated inpatient care utilizers and cost-intensive one-time inpatient care utilizers were among high-cost patients in rural China. Therefore, different strategies are needed for different utilizers to reduce the high costs. Among the top 10 chronic conditions of high-cost patients, this study found that solid tumour without metastasis, renal failure, congestive heart failure, psychoses, liver disease and diabetes (complicated) were much more prevalent among high-cost patients than non-high-cost patients.

 This study identified 31 clusters of high-cost patients primarily based on clinical characteristics through the density-based clustering algorithm OPTICS. The number of clusters identified was larger than that of a previous similar study from Powers and colleagues in the United States (10 clusters),^14^ which may result from three reasons. First, Powers and colleagues’ study focused on the high-cost Medicare Advantage population that consists of a majority of beneficiaries older than 65, while the present study included almost all of the rural residents who used healthcare. Therefore, the sample in this study may have a greater variety in clinical characteristics than the high-cost Medicare Advantage population. Second, the number of patients (n = 6154) for clustering in Powers and colleagues’ study was smaller than that in this study (n = 18 434) and they applied a priori restricted algorithm solutions to those that yielded between five and ten clusters.^18^ Third, we did not involve procedure variables in the clustering analysis due to a lack of data, potentially leading to some samples with similar characteristics in procedure variables being identified as different clusters. The similar restriction on the number of clusters was not appropriate to our sample; therefore, we relaxed the restriction. To increase operational meaning, clusters identified by OPTICS were then merged into 14 larger subgroups based on expert opinion. In addition, the number of patients who were not assigned to any cluster (15.36%) in this study was larger than that of Powers and colleagues’ study (6.21%), potentially resulting from our much larger sample size.

 This study found similar subgroups as a previous data-driven study^14^ and also had new findings which were not identified by existing studies on segmenting high-cost patients based on either data-driven methods or expert opinions.^13-15,26-28^ The chronic disease, cancer, fracture, liver disease and renal failure groups were mentioned by most previous studies, and in a recent study, the mental disease group was highlighted and listed separately from the chronic disease group.^15^ The results of the present study showed that non-trauma diseases which need surgery was the second largest subgroup including six clusters: urinary calculi, digestive system diseases (eg, appendicitis, haemorrhoids), gallstone and cholecystitis, varicosity, thyroid disorders and non-malignant hyperplasia of head, neck, ear, nose, pharynx or mouth. This subgroup seems to be unique in high-cost patients in rural China because it was seldom reported in the literature, as was the third largest subgroup, ie, the female disease group, including disease of the female reproductive system (eg, myoma of uterus) and benign breast lesions. The high costs for some of the diseases were avoidable. Urinary calculi, digestive system diseases (eg, appendicitis, haemorrhoids) and gallstones could be treated in township health centres which are primary care facilities and able to provide surgical services in rural China.^29,30^ However, patients tend to bypass primary care to seek healthcare in county hospitals or higher-level hospitals at present.^31^ Trust between primary care and patients need to be strengthened. Given that an increased burden of urolithiasis and gallstone on the healthcare system in China is anticipated,^32,33^ disease prevention is also recommended for reducing costs. Diseases of the reproductive system (eg, reproductive tract infections, cervical carcinoma) have long been more prevalent among rural women than urban women.^34-36^ More effective actions to reduce reproductive system diseases for women are needed in rural China.

 High-cost patients with renal failure had 12 admissions in 2019 and their average length of stay was 13 days. Powers and colleagues’ study reported 1.01 and 1.58 admissions for end-stage renal disease and end-stage renal disease with increased medical and behavioural comorbidity patients, respectively. Patients in the renal failure group experienced 2722 admissions in total and 83% of the admissions (2264 out of 2722) were due to uremia in 2019. In addition, 99% of the admission for uremia (2236 out of 2264) occurred in county hospitals. Dialysis centres are mainly based in large hospitals and dialysis facilities are in short supply in the community, especially in rural areas, resulting in low accessibility and high economic burden.^37,38^ Dialysis services can be transferred to outpatient settings and even primary care facilities. In 2018, the government published the standards for capacity building in delivering services for primary care facilities through a campaign called Delivering Qualified Services at Primary Care Facilities.^29^ Primary care facilities were encouraged through this campaign to construct dialysis rooms and provide dialysis services. The average length of stay among high-cost patients with mental disease was high (32.11 days). These groups of patients were represented by schizophrenia, which is a severe mental illness. A systematic review was published in the Cochrane Database of Systematic Reviews and designed to evaluate the effect of short or brief admissions (defined as less than 28 days) on hospital care for persons with serious mental illness compared with longer-stay hospital admissions. This review found that short-stay hospitalization did not lead to poor or fragmented care and short-stay patients possibly had a greater chance of finding employment.^39,40^ Shorter stays lead to lower spending, and whether short-stay hospitalization fitting in severe mental health patients in China needs further analysis.

 The proportion of PICs on total spending varied considerably among different subgroups. Priority may need to be given to the chronic disease group, of which PICs accounted for more than 10% of total spending. For the chronic disease group, the proportion of PIC on total spending (10.57%) was higher than that of Powers and colleagues’ study, potentially resulting from different chronic disease composition or because patients were more likely to occur preventable hospitalizations in rural China.^10,20^ The PIC of some of the subgroups only amounted to 1%–2% of total spending (including the female disease group, cancer group, fracture group, and renal failure group), indicating the limited ability to cut down healthcare spending through lowering preventable hospitalizations for these groups of high-cost patients. Though the limited ability to cut down healthcare spending through lowering preventable hospitalizations for cancer group, studies showed expanding role of primary care in cancer control.^41,42^ The strengths of primary care (eg, continuous, coordinated, and comprehensive care) are particularly evident in prevention and diagnosis, in shared follow-up and survivorship care, and in end-of-life care. This needs to be realised by policy-maker, health insures, and providers in China. Previous study showed that liver and neurologic subgroups had persistently high spending and the spending mainly came from prescription drug costs, implying that the rational use and pricing of specialty pharmaceuticals may be effective strategies for reducing spending,^14^ which needs further evidence in China.

 Though some of the subgroups were only related to single body system (eye disease, skin disease), it may be difficult to design one-size-fit-all intervention for all of the patients within one subgroup. For example, eye disease subgroup was represented by patients with cataract in this study. However, around 25% of these patients sought care in large hospitals outside the county and experienced one-time high spending, while the rest patients were usually with chronic conditions (eg, diabetes) which added to their spending. For subgroups dominated by acute events (fracture, respiratory infection/inflammation), the opportunities to reduce spending may be limited.^14^

 This study has strengths and limitations. This study broadens our understanding of subgroups of high-cost patients from non-high-income countries. However, we did not include procedure and functional status variables in the clustering analysis due to a lack of data. Further study with more comprehensive variables is needed. In this study, we were able to identify patients with surgery through the department variable, though without procedure variables. Moreover, with only principal diagnosis available to define PPHs, we did not exclude patients with severe complications or comorbidities. Given that previous research from rural China showed that the proportion of exclusion admissions in PPHs was smaller than 0.8%, we believe that the overestimation of the number of PPHs was minor in the present work. No existing tool was developed in the context of China, so PPHs were identified according to the algorithm developed for the United States. Although this algorithm was validated in previous studies from China, we still recommend the development of a tool for China.

## Conclusion

 High-cost patients were ten years older and more likely to be from poverty-stricken families than non-high-cost patients, with 2.47 admissions and 3.25 outpatient visits annually. High-cost patients in rural China were classified into 14 clinically distinct subgroups which had different healthcare utilization and spending characteristics. Non-trauma diseases which need surgery and the female disease group were the second and third largest subgroups, respectively, which seem to be unique in the high-cost population in rural China. The high costs for non-trauma diseases which need surgery were avoidable if primary care could be well-utilized, and more effective actions to reduce reproductive system diseases for women are needed in rural China. Dialysis services can be transferred to outpatient settings and even primary care facilities to increase accessibility and reduce the economic burden for high-cost patients with renal failure. The proportion of PICs on total spending varied a lot among different subgroups. Priority could be given to developing strategies for the chronic disease group, of which PICs accounted for more than 10% of total spending. However, the ability to cut down healthcare spending through lowering preventable hospitalizations is limited for certain subgroups with low preventable spending (eg, female disease, cancer, fracture, and renal failure groups).

## Acknowledgements

 We thank our colleagues for their effort in the data collection and the local site for its participation in the investigation.

## Ethical issues

 The study was approved by the Ethics Committee of Tongji Medical College, Huazhong University of Science and Technology. All methods were performed in accordance with the relevant guidelines and regulations.

## Competing interests

 Authors declare that they have no competing interests.

## Funding

 This work was supported by the National Natural Science Foundation of China (Grant no: 72104086). The organization had no role in the study design, data collection, analysis, interpretation, and in writing the manuscript.

## Supplementary files


Supplementary file 1. A Full Description of Study Variables.

Supplementary file 2. The List of the 187 ADRGs and the 31 Groups of Chronic Conditions; The List of Variables Used for Clustering.

Supplementary file 3. The Results of Algorithms Performance Evaluation.

Supplementary file 4. The List of ICD-10 Codes Used to Identify PPHs.

Supplementary file 5. The Healthcare Utilization and Spending for the 31 Clusters.

## References

[1] Global Burden of Disease 2020 Health Financing Collaborator Network. Tracking development assistance for health and for COVID-19: a review of development assistance, government, out-of-pocket, and other private Lu et al International Journal of Health Policy and Management, 2024;13:8151 11 spending on health for 204 countries and territories, 1990-2050. Lancet. 2021;398(10308):1317-1343. 10.1016/s0140-6736(21)01258-7 PMC8457757 34562388

[2] World Health Organization (WHO). Global Spending on Health: Weathering the Storm. https://www.who.int/publications-detail-redirect/9789240017788. Accessed December 29, 2022.

[3] McKee M, Stuckler D (2020). If the world fails to protect the economy, COVID-19 will damage health not just now but also in the future. Nat Med.

[4] Hayes SL, Salzberg CA, McCarthy D (2016). High-need, high-cost patients: who are they and how do they use health care? A population-based comparison of demographics, health care use, and expenditures. Issue Brief (Commonw Fund).

[5] Blumenthal D, Chernof B, Fulmer T, Lumpkin J, Selberg J (2016). Caring for high-need, high-cost patients - an urgent priority. N Engl J Med.

[6] Joynt KE, Gawande AA, Orav EJ, Jha AK (2013). Contribution of preventable acute care spending to total spending for high-cost Medicare patients. JAMA.

[7] Wammes JJG, van der Wees PJ, Tanke MAC, Westert GP, Jeurissen PPT (2018). Systematic review of high-cost patients’ characteristics and healthcare utilisation. BMJ Open.

[8] Zook CJ, Moore FD (1980). High-cost users of medical care. N Engl J Med.

[9] Zhang Y, Lu S, Niu Y, Zhang L (2018). Medical expenditure clustering and determinants of the annual medical expenditures of residents: a population-based retrospective study from rural China. BMJ Open.

[10] Lu S, Zhang Y, Zhang L, Klazinga NS, Kringos DS (2022). Characterizing potentially preventable hospitalizations of high-cost patients in rural China. Front Public Health.

[11] Figueroa JF, Joynt Maddox KE, Beaulieu N, Wild RC, Jha AK (2017). Concentration of potentially preventable spending among high-cost Medicare subpopulations: an observational study. Ann Intern Med.

[12] Graven PF, Meath TH, Mendelson A, Chan BK, Dorr DA, McConnell KJ (2016). Preventable acute care spending for high-cost patients across payer types. J Health Care Finance.

[13] Joynt KE, Figueroa JF, Beaulieu N, Wild RC, Orav EJ, Jha AK (2017). Segmenting high-cost Medicare patients into potentially actionable cohorts. Healthc (Amst).

[14] Powers BW, Yan J, Zhu J (2019). Subgroups of high-cost Medicare advantage patients: an observational study. J Gen Intern Med.

[15] Zhang Y, Grinspan Z, Khullar D (2020). Developing an actionable patient taxonomy to understand and characterize high-cost Medicare patients. Healthc (Amst).

[16] Lee NS, Whitman N, Vakharia N, Taksler GB, Rothberg MB (2017). High-cost patients: hot-spotters don’t explain the half of it. J Gen Intern Med.

[17] Khullar D, Zhang Y, Kaushal R (2020). Potentially preventable spending among high-cost Medicare patients: implications for healthcare delivery. J Gen Intern Med.

[18] Yan J, Linn KA, Powers BW (2019). Applying machine learning algorithms to segment high-cost patient populations. J Gen Intern Med.

[19] Agency for Healthcare Research and Quality. Prevention Quality Indicators Overview. https://www.qualityindicators.ahrq.gov/Modules/pqi_resources.aspx. Accessed January 5, 2021.

[20] Chen T, Pan J (2022). The effect of spatial access to primary care on potentially avoidable hospitalizations of the elderly: evidence from Chishui city, China. Soc Indic Res.

[21] Rosella LC, Fitzpatrick T, Wodchis WP, Calzavara A, Manson H, Goel V (2014). High-cost health care users in Ontario, Canada: demographic, socio-economic, and health status characteristics. BMC Health Serv Res.

[22] Wammes JJG, Tanke M, Jonkers W, Westert GP, Van der Wees P, Jeurissen PP (2017). Characteristics and healthcare utilisation patterns of high-cost beneficiaries in the Netherlands: a cross-sectional claims database study. BMJ Open.

[23] Zhang L, Han R (2020). Does poverty reduce the life expectancy of the elderly: research on multistate life table. Chin J Public Health.

[24] Xie F, Zhu Z (2018). Resources and utilizations of medical services in poverty-stricken areas in China. Chin J Public Health.

[25] Fitzpatrick T, Rosella LC, Calzavara A (2015). Looking beyond income and education: socioeconomic status gradients among future high-cost users of health care. Am J Prev Med.

[26] Long P, Abrams M, Milstein A, Andersen G, Apton K, Dahlberg M. Effective Care for High-Need Patients: Opportunities for Improving Outcomes, Value and Health. Washington, DC: National Academy of Medicine; 2017. 37748007

[27] Clough JD, Riley GF, Cohen M (2016). Patterns of care for clinically distinct segments of high-cost Medicare beneficiaries. Healthc (Amst).

[28] Johnson TL, Brewer D, Estacio R (2015). Augmenting predictive modeling tools with clinical insights for care coordination program design and implementation. EGEMS (Wash DC).

[29] National Health Commission, State Administration of Traditional Chinese Medicine. Notice of the National Health Commission and the State Administration of Traditional Chinese Medicine on Carrying out the “Delivering Qualified Services at Primary Care Facilities” Campaign. http://www.gov.cn/zhengce/zhengceku/2018-12/31/content_5435449.htm. Accessed April 23, 2023.

[30] Li Z, Yang J, Wu Y (2018). Challenges for the surgical capacity building of township hospitals among the Central China: a retrospective study. Int J Equity Health.

[31] Lu S, Li Y, Gao H, Zhang Y (2022). Difference in bypass for inpatient care and its determinants between rural and urban residents in China. Int J Equity Health.

[32] Wang Q, Wang Y, Yang C (2023). Trends of urolithiasis in China: a national study based on hospitalized patients from 2013 to 2018. Kidney Dis (Basel).

[33] Su Z, Gong Y, Liang Z (2020). Prevalence of gallstone in Mainland China: a meta-analysis of cross-sectional studies. Clin Res Hepatol Gastroenterol.

[34] Cao J (2010). Reasons for the high incidence of reproductive system diseases among rural women and their prevention and control strategies. Contemp Med.

[35] Wang Y, Li Y, Wu Y, Feng Y, Du J (2019). Analysis of screening results for reproductive system and breast diseases in 12000 women of reproductive age in Chengde city. Hebei Medicine.

[36] Yang LR, Zhao H, Wang HP (2006). Improving ability of married women to prevent reproductive tract infections in rural western China. Environ Health Prev Med.

[37] Su F, Wang J, Zhang L, Lv Y (2021). Central dialysate supply system+medical alliance to promote the development of community dialysis centers. Journal of China-Japan Friendship Hospital.

[38] Li Y, Wang M (2013). The challenge and measures after dialysis therapy entered in rural medical insurance of serious diseases. Chin Health Econ.

[39] Babalola O, Gormez V, Alwan NA, Johnstone P, Sampson S (2014). Length of hospitalisation for people with severe mental illness. Cochrane Database Syst Rev.

[40] Weeks SM, Giles K (2015). Length of stay in hospital for people with severe mental illness. Int J Evid Based Healthc.

[41] Rubin G, Berendsen A, Crawford SM (2015). The expanding role of primary care in cancer control. Lancet Oncol.

[42] Usher-Smith J, Emery J, Hamilton W, Griffin SJ, Walter FM (2015). Risk prediction tools for cancer in primary care. Br J Cancer.

