# Supplementary material for: Subgroups of High-Cost Patients and Their Preventable Inpatient Cost in Rural China
Source: Int J Health Policy Manag. 2024 Mar 9;13:8151. doi: 10.34172/ijhpm.2024.8151 (PMC11608279; doi:10.34172/ijhpm.2024.8151)
Supplement: Supplementary file 1 — A Full Description of Study Variables. [file ijhpm-13-8151-s001.pdf]

Article title: Subgroups of High-Cost Patients and Their Preventable Inpatient Cost in Rural China

Journal name: International Journal of Health Policy and Management (IJHPM)

Authors' information: Shan Lu[1,2,] Yan Zhang[1,2], Ting Ye[1,2]*, Dionne S. Kringos[3]

[1]School of Medicine and Health Management, Tongji Medical College, Huazhong University of Science and Technology, Wuhan, China.

[2]Research Centre for Rural Health Service, Key Research Institute of Humanities & Social Sciences of Hubei Provincial Department of Education, Wuhan, China.

[3]Amsterdam Public Health Research Institute, Department of Public and Occupational Health, University of Amsterdam, Amsterdam UMC, Amsterdam, The Netherlands.

***Correspondence to:** Ting Ye; Email: yeting@hust.edu.cn

Citation: Lu S, Zhang Y, Ye T, Kringos DS. Subgroups of high-cost patients and their preventable inpatient cost in rural China. Int J Health Policy Manag. 2024;13:8151. doi:10.34172/ijhpm.2024.8151

Supplementary file 1. A Full Description of Study Variables

Table S1 A description of study variables

Variables	Definitions and categories
Demographics	
Age*	Age of the patient (continuous)
Gender*	Gender of the patient<br>0=female, 1=male
Economic status	
Family income	Whether or not the patient is from a poverty-stricken family according to the Poverty Alleviation Information System<br>0=non- poverty-stricken family, 1= poverty-stricken family
Clinical characteristics[#]	
Active diagnosis*	Principle diagnosis for each outpatient visit or admission in 2019, categorized into 187 groups
Chronic conditions*	Chronic conditions of the patients, categorized into 31 groups
Departments*	Department where the patients were hospitalized in 2019
Health care utilization	
Admissions	Hospitalization frequency of the patient during 2019
Admissions_within_county	Number of admissions within the county of the patient during 2019
Admissions_outside_county	Number of admissions outside the county of the patient during 2019
Average LOS	Average length of stay of the patient (day)
Visits	Number of outpatient visits of the patient during 2019
Visits_within_county	Number of outpatient visits within the county of the patient during 2019
Visits_outside_county	Number of outpatient visits outside the county of the patient during 2019
Spending characteristics	
Total spending	Total annual inpatient and outpatient spending of the patient during 2019
Preventable inpatient spending	Preventable inpatient spending of the patient during 2019

* Variables for clustering analysis.

[#] Active diagnosis, chronic conditions and departments were classified into 187, 31 and 9 groups respectively, and we included each group as a binary variable (i.e. 0-1 variable) in Clustering algorithm with 1 representing the existence of an active diagnosis, a chronic condition or a department for the patient.
