# Supplementary material for: Subgroups of High-Cost Patients and Their Preventable Inpatient Cost in Rural China
Source: Int J Health Policy Manag. 2024 Mar 9;13:8151. doi: 10.34172/ijhpm.2024.8151 (PMC11608279; doi:10.34172/ijhpm.2024.8151)
Supplement: Supplementary file 2 — The List of the 187 ADRGs and the 31 Groups of Chronic Conditions; The List of Variables Used for Clustering. [file ijhpm-13-8151-s002.pdf]

Article title: Subgroups of High-Cost Patients and Their Preventable Inpatient Cost in Rural China

Journal name: International Journal of Health Policy and Management (IJHPM)

Authors' information: Shan Lu[1,2,] Yan Zhang[1,2], Ting Ye[1,2]*, Dionne S. Kringos[3]

[1]School of Medicine and Health Management, Tongji Medical College, Huazhong University of Science and Technology, Wuhan, China.

[2]Research Centre for Rural Health Service, Key Research Institute of Humanities & Social Sciences of Hubei Provincial Department of Education, Wuhan, China.

[3]Amsterdam Public Health Research Institute, Department of Public and Occupational Health, University of Amsterdam, Amsterdam UMC, Amsterdam, The Netherlands.

***Correspondence to:** Ting Ye; Email: yeting@hust.edu.cn

Citation: Lu S, Zhang Y, Ye T, Kringos DS. Subgroups of high-cost patients and their preventable inpatient cost in rural China. Int J Health Policy Manag. 2024;13:8151. doi:10.34172/ijhpm.2024.8151

Supplementary file 2. The List of the 187 ADRGs and the 31 Groups of Chronic Conditions; The List of Variables Used for Clustering

Table S1 The list of the 187 ADRGs

Code	Description
ADRG1	Intracranial haemorrhage
ADRG2	Cerebral ischemic disease
ADRG3	Non traumatic consciousness disorder
ADRG4	Viral brain, spinal cord and meningitis
ADRG5	Other infections of the nervous system
ADRG6	Neurologic tumour
ADRG7	Neurodegenerative disorders
ADRG8	Demyelination and cerebellar ataxia
ADRG9	Epilepsy
ADRG10	Neuromuscular disease
ADRG11	headache
ADRG12	Congenital diseases of nervous system
ADRG13	Cerebral palsy
ADRG14	Brain dysfunction
ADRG15	Cranial / peripheral nerve disorders
ADRG16	open brain injury
ADRG17	Closed brain injury
ADRG18	Spinal cord injury and dysfunction
ADRG19	Other neurological disorders
ADRG20	Malignant tumour of eye and borderline tumour
ADRG21	Neurovascular diseases in eyes
ADRG22	Non operative treatment of anterior chamber haemorrhage and ocular trauma
ADRG23	Acute major eye infection
ADRG24	Various types of glaucoma
ADRG25	Cataract of various types
ADRG26	Other diseases cause eye lesions
ADRG27	Other eye diseases
ADRG28	Malignant tumours of head, neck, ear, nose, pharynx and mouth
ADRG29	Imbalance and hearing disorders
ADRG30	Upper respiratory tract infection and tympanitis
ADRG31	Epiglottis, laryngitis and tracheitis
ADRG32	Trauma and deformation of head, neck, external ear, mouth and nose
ADRG33	Head, neck, ear, nose, pharynx and mouth are non malignant proliferative
ADRG34	Oral and dental related diseases
ADRG35	Other head, neck, ear, nose, pharyngeal, mouth diseases
ADRG36	Respiratory system tumour
ADRG37	pulmonary embolism
ADRG38	Pulmonary edema and respiratory failure
ADRG39	Tuberculosis of respiratory system
ADRG40	Respiratory infection / inflammation
ADRG41	Pulmonary interstitial disease
ADRG42	Chronic obstructive airway disease
ADRG43	Major chest trauma

ADRG44	Respiratory symptoms and signs
ADRG45	Pleural lesions and pleural effusion
ADRG46	Asthma and asthmatic bronchitis
ADRG47	Pertussis and acute bronchitis
ADRG48	Other respiratory disorders
ADRG49	Heart failure, shock
ADRG50	acute myocardial infarction
ADRG51	angina pectoris
ADRG52	Coronary atherosclerosis / thrombus / occlusion
ADRG53	Circulatory system tumour
ADRG54	Cardiomyopathy
ADRG55	Infective endocarditis
ADRG56	Valve disease
ADRG57	Severe arrhythmia and cardiac arrest
ADRG58	Arrhythmia and conduction disorder
ADRG59	Congenital heart disease
ADRG60	hypertension
ADRG61	Syncope and / or vanity
ADRG62	Chest pain
ADRG63	Arterial disease
ADRG64	Venous disease
ADRG65	Other circulatory system disorders
ADRG66	Digestive system malignant tumour
ADRG67	Gastrointestinal bleeding
ADRG68	Inflammatory bowel disease
ADRG69	Peptic ulcer with bleeding or perforation
ADRG70	Other peptic ulcer
ADRG71	Obstruction of digestive tract or abdominal pain
ADRG72	Esophagitis, gastroenteritis
ADRG73	Other digestive system diagnosis
ADRG74	Hepatobiliary pancreatic system malignant tumour
ADRG75	Liver failure
ADRG76	cirrhosis
ADRG77	Viral hepatitis
ADRG78	acute pancreatitis
ADRG79	Acute biliary tract disease
ADRG80	Other liver diseases
ADRG81	Other diseases of biliary tract
ADRG82	Other pancreatic diseases
ADRG83	Pelvic fracture
ADRG84	Femoral neck fracture
ADRG85	Fracture of femoral shaft and distal end
ADRG86	Injury to forearm, wrist, hand or foot
ADRG87	Injury except forearm, wrist, hand and foot
ADRG88	osteomyelitis

ADRG89	Chronic inflammatory musculoskeletal connective tissue disease
ADRG90	Infectious arthritis
ADRG91	Osteopathy and other joint diseases
ADRG92	Neck and back disease
ADRG93	Malignant lesions and diseases of bone, muscle and connective tissue
ADRG94	Congenital skeletal and muscular diseases except spine
ADRG95	The rehabilitation of musculoskeletal implant / prosthesis
ADRG96	Other bones, muscles, tendons, connective tissue
ADRG97	Breast cancer
ADRG98	Malignant tumour of skin and subcutaneous tissue
ADRG99	Major skin disorders
ADRG100	Inflammatory dermatosis
ADRG101	Trauma of breast, skin and subcutaneous tissue
ADRG102	Infectious dermatosis
ADRG103	Non malignant proliferative lesions of skin and subcutaneous tissue
ADRG104	Benign breast lesions
ADRG105	Other skin and breast diseases
ADRG106	Endocrine gland malignant tumour
ADRG107	Diabetes
ADRG108	Endocrine disorders
ADRG109	Dystrophic
ADRG110	Congenital metabolic abnormality
ADRG111	Other metabolic disorders
ADRG112	Renal insufficiency
ADRG113	Nephritis and nephrosis
ADRG114	Renal and urinary tract tumours
ADRG115	Renal and urinary tract infection
ADRG116	Hypertension / diabetic nephropathy
ADRG117	Signs and symptoms of kidney and urinary tract
ADRG118	Urinary calculi, obstruction and urethral stricture
ADRG119	Renal and urinary tract injury
ADRG120	Other diseases of kidney and urinary system
ADRG121	Male reproductive system malignant tumour
ADRG122	Inflammation of male reproductive system
ADRG123	Other male reproductive system disorders
ADRG124	Malignant tumour of female reproductive system
ADRG125	Female reproductive infection
ADRG126	Other diseases of female reproductive system
ADRG127	Vaginal delivery
ADRG128	Puerperal related diseases
ADRG129	Abortion related diseases
ADRG130	Ectopic pregnancy
ADRG131	Other pregnancy related diseases
ADRG132	Neonatal respiratory distress syndrome
ADRG133	Extreme stunt (birth weight < 1500g)

ADRG134	Premature infants (birth weight 1500-2499g)
ADRG135	Premature infants (birth weight > 2499g)
ADRG136	Term infants
ADRG137	From newborn (29 days ≤ birth age < 1 year old)
ADRG138	Reticuloendothelium and immune diseases
ADRG139	Erythrocytic disease and nutritional anaemia
ADRG140	Hemolytic anaemia
ADRG141	Aplastic anaemia
ADRG142	Other anaemia
ADRG143	coagulation disorders
ADRG144	acute leukaemia
ADRG145	Lymphoma and other types of leukaemia
ADRG146	Myeloma
ADRG147	Non-specific malignant tumour
ADRG148	Non-specific benign tumour
ADRG149	Chemistry and / or target, and growth of malignant proliferative diseases
ADRG150	Immunotherapy for malignant proliferative diseases
ADRG151	Radiotherapy for malignant proliferative diseases
ADRG152	Follow up examination after treatment of malignant proliferative diseases
ADRG153	Maintenance treatment of malignant proliferative diseases
ADRG154	Septicaemia
ADRG155	Post operation and post-traumatic infection
ADRG156	Fever with unknown cause
ADRG157	Viral disease
ADRG158	Bacterial disease
ADRG159	Other infectious or parasitic diseases
ADRG160	Schizophrenia
ADRG161	Paranoia and acute psychosis
ADRG162	Major emotional barriers
ADRG163	Neurotic disorders and other affective disorders
ADRG164	Eating and sleep disorders
ADRG165	personality disorder
ADRG166	Mental development disorder in childhood
ADRG167	Anxiety disorder
ADRG168	Organic and symptomatic mental disorders
ADRG169	Alcoholism and rehabilitation
ADRG170	Doping abuse and dependence
ADRG171	damage
ADRG172	Allergic reaction
ADRG173	Drug poisoning or toxic reaction
ADRG174	Medical sequelae
ADRG175	Other injuries, poisoning and toxic reactions
ADRG176	Third degree burns for more than 30% of the body surface or more
ADRG177	Other burns, corrosion injuries and frostbite
ADRG178	recovery

ADRG179	Other rehabilitation treatment
ADRG180	Signs and symptom
ADRG181	Follow up (excluding malignant tumour diagnosis)
ADRG182	Other later care
ADRG183	Non-specific congenital malformation
ADRG184	Other factors affecting health status
ADRG185	HIV related diseases
ADRG186	Other HIV related information
ADRG187	Multiple severe trauma, no operation

Table S2 The list of the 31 groups of chronic conditions

Code	Description
Chronic1	Congestive Heart Failure
Chronic2	Cardiac arrhythmias
Chronic3	Valvular Disease
Chronic4	Pulmonary Circulation Disorders
Chronic5	Peripheral Vascular Disorders
Chronic6	Hypertension, uncomplicated
Chronic7	Hypertension, complicated
Chronic8	Paralysis
Chronic9	Other Neurological Disorders
Chronic10	Chronic Pulmonary Disease
Chronic11	Diabetes Uncomplicated
Chronic12	Diabetes Complicated
Chronic13	Hypothyroidism
Chronic14	Renal Failure
Chronic15	Liver Disease
Chronic16	Peptic Ulcer Disease excluding bleeding
Chronic17	AIDS/HIV
Chronic18	Lymphoma
Chronic19	Metastatic Cancer
Chronic20	Solid Tumor without Metastasis
Chronic21	Rheumatoid Arthritis/collagen
Chronic22	Coagulopathy
Chronic23	Obesity
Chronic24	Weight Loss
Chronic25	Fluid and Electrolyte Disorders
Chronic26	Blood Loss Anemia
Chronic27	Deficiency Anemia
Chronic28	Alcohol Abuse
Chronic29	Drug Abuse
Chronic30	Psychoses
Chronic31	Depression

Table S3 The List of Variables Used for Clustering

Code	Description
ADRG1	Intracranial haemorrhage
ADRG2	Cerebral ischemic disease
ADRG11	headache
ADRG19	Other neurological disorders
ADRG25	Cataract of various types
ADRG27	Other eye diseases
ADRG29	Imbalance and hearing disorders
ADRG30	Upper respiratory tract infection and tympanitis
ADRG33	Head, neck, ear, nose, pharynx and mouth are non malignant proliferative
ADRG34	Oral and dental related diseases
ADRG35	Other head, neck, ear, nose, pharyngeal, mouth diseases
ADRG36	Respiratory system tumour
ADRG40	Respiratory infection / inflammation
ADRG42	Chronic obstructive airway disease
ADRG44	Respiratory symptoms and signs
ADRG46	Asthma and asthmatic bronchitis
ADRG49	Heart failure, shock
ADRG51	angina pectoris
ADRG52	Coronary atherosclerosis / thrombus / occlusion
ADRG58	Arrhythmia and conduction disorder
ADRG60	hypertension
ADRG62	Chest pain
ADRG64	Venous disease
ADRG65	Other circulatory system disorders
ADRG66	Digestive system malignant tumour
ADRG71	Obstruction of digestive tract or abdominal pain
ADRG72	Esophagitis, gastroenteritis
ADRG73	Other digestive system diagnosis
ADRG79	Acute biliary tract disease
ADRG81	Other diseases of biliary tract
ADRG86	Injury to forearm, wrist, hand or foot
ADRG87	Injury except forearm, wrist, hand and foot
ADRG91	Osteopathy and other joint diseases
ADRG92	Neck and back disease
ADRG96	Other bones, muscles, tendons, connective tissue
ADRG99	Major skin disorders
ADRG100	Inflammatory dermatosis
ADRG104	Benign breast lesions
ADRG107	Diabetes
ADRG108	Endocrine disorders
ADRG110	Congenital metabolic abnormality
ADRG112	Renal insufficiency
ADRG113	Nephritis and nephrosis

ADRG115	Renal and urinary tract infection
ADRG118	Urinary calculi, obstruction and urethral stricture
ADRG120	Other diseases of kidney and urinary system
ADRG123	Other male reproductive system disorders
ADRG125	Female reproductive infection
ADRG126	Other diseases of female reproductive system
ADRG131	Other pregnancy related diseases
ADRG137	From newborn (29 days $\leq$ birth age < 1 year old)
ADRG151	Radiotherapy for malignant proliferative diseases
ADRG156	Fever with unknown cause
ADRG160	Schizophrenia
ADRG179	Other rehabilitation treatment
ADRG180	Signs and symptom
ADRG181	Follow up (excluding malignant tumour diagnosis)
ADRG184	Other factors affecting health status
Chronic1	Congestive Heart Failure
Chronic2	Cardiac arrhythmias
Chronic3	Valvular Disease
Chronic5	Peripheral Vascular Disorders
Chronic6	Hypertension, uncomplicated
Chronic7	Hypertension, complicated
Chronic8	Paralysis
Chronic9	Other Neurological Disorders
Chronic10	Chronic Pulmonary Disease
Chronic11	Diabetes Uncomplicated
Chronic12	Diabetes Complicated
Chronic14	Renal Failure
Chronic15	Liver Disease
Chronic16	Peptic Ulcer Disease excluding bleeding
Chronic18	Lymphoma
Chronic19	Metastatic Cancer
Chronic20	Solid Tumor without Metastasis
Chronic21	Rheumatoid Arthritis/collagen
Chronic22	Coagulopathy
Chronic24	Weight Loss
Chronic25	Fluid and Electrolyte Disorders
Chronic27	Deficiency Anemia
Chronic30	Psychoses
Chronic31	Depression
Department1	Internal medicine
Department2	Surgery
Department3	Orthopedics
Department4	Gynaecology
Department5	Obstetrics
Department6	Paediatrics

Department7	Oncology
Department8	Ophthalmology & ENT & stomatology
Department9	Rehabilitation
