# Supplementary material for: Subgroups of High-Cost Patients and Their Preventable Inpatient Cost in Rural China
Source: Int J Health Policy Manag. 2024 Mar 9;13:8151. doi: 10.34172/ijhpm.2024.8151 (PMC11608279; doi:10.34172/ijhpm.2024.8151)
Supplement: Supplementary file 3 — The Results of Algorithms Performance Evaluation. [file ijhpm-13-8151-s003.pdf]

Article title: Subgroups of High-Cost Patients and Their Preventable Inpatient Cost in Rural China

Journal name: International Journal of Health Policy and Management (IJHPM)

Authors' information: Shan Lu[1,2,] Yan Zhang[1,2], Ting Ye[1,2]*, Dionne S. Kringos[3]

[1]School of Medicine and Health Management, Tongji Medical College, Huazhong University of Science and Technology, Wuhan, China.

[2]Research Centre for Rural Health Service, Key Research Institute of Humanities & Social Sciences of Hubei Provincial Department of Education, Wuhan, China.

[3]Amsterdam Public Health Research Institute, Department of Public and Occupational Health, University of Amsterdam, Amsterdam UMC, Amsterdam, The Netherlands.

***Correspondence to:** Ting Ye; Email: yeting@hust.edu.cn

Citation: Lu S, Zhang Y, Ye T, Kringos DS. Subgroups of high-cost patients and their preventable inpatient cost in rural China. Int J Health Policy Manag. 2024;13:8151. doi:10.34172/ijhpm.2024.8151

Supplementary file 3. The Results of Algorithms Performance Evaluation

(a) The visual examination of the cluster assignments:

For Density-Based clustering, since different tuning parameters can lead to the same number of clusters, we extracted 229 solutions from the OPTICS algorithm by varying the radius ε from 4.02 to 6.30 in increments of 0.01. This range of radii was able to capture all the clustering solutions that produced 5 or more clusters. For centroid-based clustering, we computed 46 distinct solutions, letting k range from 5 to 50. For connectivity-based clustering, we found the optimal tuning parameters that provided 46 distinct solutions with 5 to 50 clusters. (For centroid-based and connectivity-based clustering, we needed to a priori restrict the number of clusters, so we restricted the number of clusters within a relatively wide range, i.e. from 5 to 50.) We then calculated and compared the average silhouette width for each solution and selected the solution with the highest average silhouette width following Yan and colleagues' research[1].

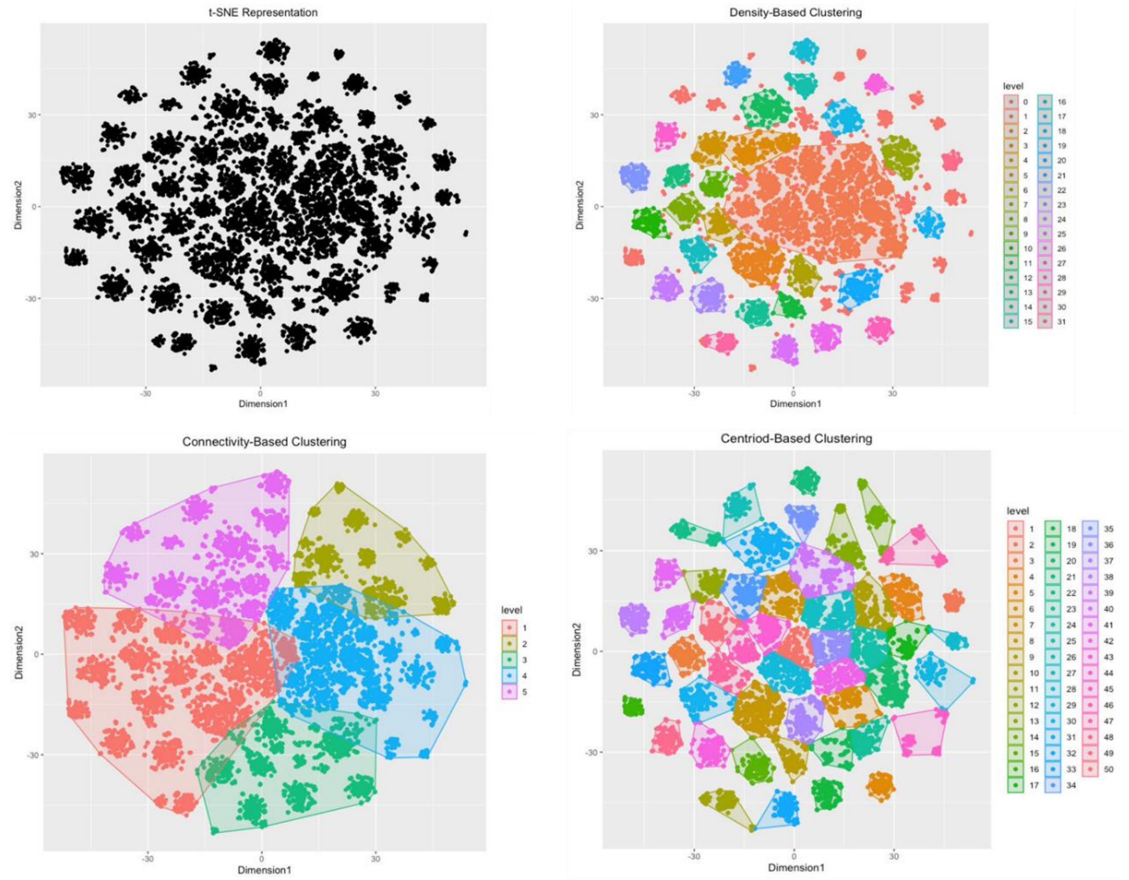

Figure S1 Visual representation of patient clusters, by clustering method

(The figures represent two-dimensional projection of our dataset generated by the t-SNE dimension reduction algorithm. Each point is a patient in the study population, and the distance between two points approximates their similarity in high-dimensional space. The top left plot represents the t-SNE projection in insolation. The remaining plots overlay colored convex cluster outlines to the t-SNE projection for each of the three clustering methods.)

(b) The results of the ridge regression analysis:

We followed Yan and colleagues' research[1] to conduct a set of ridge regression models in order to better

understand the relationship between cluster assignment and clinical variables. A separate set of models was implemented for each cluster. The dependent variable in each model was a dichotomous indicator of assignment to a given cluster, and the original 91 variables were independent variables. The magnitudes of the resulting model coefficient estimates represent the relative contribution of each independent variable to discriminating patients in one cluster from other high-cost patients.

For ridge regression model fitting, multiple separate models were implemented for each cluster with the dependent variable being a dichotomous indicator of assignment to a given cluster. More specifically, for a given cluster assignment derived from the OPTICS cluster analysis, we fit 100 ridge-penalized logistic regressions, where the binary outcome in each regression was the indicator of membership in the group with that particular cluster assignment label. The 100 regressions corresponded to 100 values of the ridge penalty parameter, which effectively reduces correlation amongst the predictor variables and leads to a more stable solution. The ridge regression estimated coefficient ranges in Figure S2 were computed after averaging the estimated model coefficients over the 100 fitted models. Each range was calculated as the maximum minus the minimum value. This metric is a measure of variable importance, where a large value of the range suggests that the variable is useful for distinguishing observations in one cluster from the rest. To mitigate non-comparability of the estimated coefficients across models, we specified the same set of ridge penalties for all regression models within and across all three clustering methods. In addition, all variables in the data set were standardized to have mean zero and unit variance before model fitting.

Following model fitting, we computed the range of the estimated coefficients across clusters for each independent variable. In order to visualize these results, we plotted the values of the variables with the largest 20 ranges for each of the clustering methods following Yan and colleagues' study[1].

The results of the ridge regression analysis are shown in Figure S2, which presents the estimated coefficient ranges for the 20 variables with the ranges among each clustering method. This range roughly demonstrates the extent to which the variable contributes to differentiation among clusters. Density-based clustering had a larger range for the 14 highest ranked variables, and a lightly smaller range than that of the Centroid-based clustering for the following 4 variables, after which, the two methods converged.

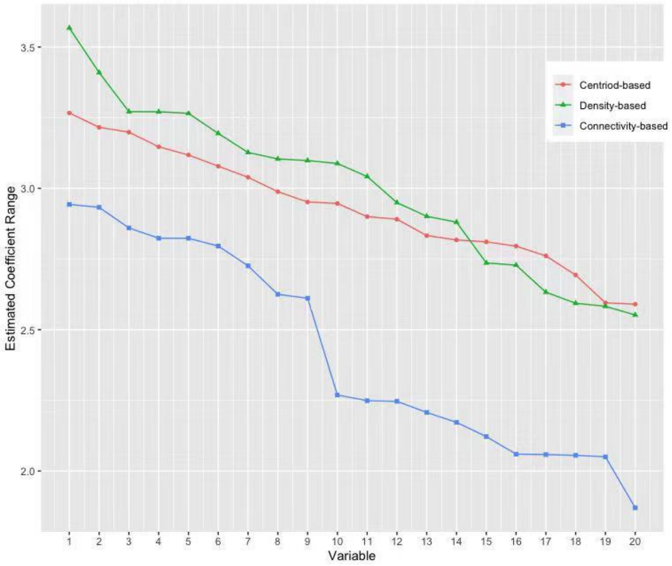

Figure S2 Summary of the results of the ridge regression analysis

References:

1 Yan J, Linn KA, Powers BW, et al. Applying Machine Learning Algorithms to Segment High-Cost Patient Populations. J Gen Intern Med 2019;34:211–17.
