# Supplementary material for: Subgroups of High-Cost Patients and Their Preventable Inpatient Cost in Rural China
Source: Int J Health Policy Manag. 2024 Mar 9;13:8151. doi: 10.34172/ijhpm.2024.8151 (PMC11608279; doi:10.34172/ijhpm.2024.8151)
Supplement: Supplementary file 4 — The List of ICD-10 Codes Used to Identify PPHs. [file ijhpm-13-8151-s004.pdf]

Article title: Subgroups of High-Cost Patients and Their Preventable Inpatient Cost in Rural China

Journal name: International Journal of Health Policy and Management (IJHPM)

Authors' information: Shan Lu[1,2,] Yan Zhang[1,2], Ting Ye[1,2]*, Dionne S. Kringos[3]

[1]School of Medicine and Health Management, Tongji Medical College, Huazhong University of Science and Technology, Wuhan, China.

[2]Research Centre for Rural Health Service, Key Research Institute of Humanities & Social Sciences of Hubei Provincial Department of Education, Wuhan, China.

[3]Amsterdam Public Health Research Institute, Department of Public and Occupational Health, University of Amsterdam, Amsterdam UMC, Amsterdam, The Netherlands.

***Correspondence to:** Ting Ye; Email: yeting@hust.edu.cn

Citation: Lu S, Zhang Y, Ye T, Kringos DS. Subgroups of high-cost patients and their preventable inpatient cost in rural China. Int J Health Policy Manag. 2024;13:8151. doi:10.34172/ijhpm.2024.8151

Supplementary file 4. The List of ICD-10 Codes Used to Identify PPHs

Table S1 List of ICD-10 codes used to identify potentially preventable hospitalizations

Category	Inclusions for principal diagnosis	Exclusions for any listed diagnosis
Diabetes short-term complications	E10.0 E10.1 E11.0 E11.1	
Diabetes long-term complications	E10.2-E10.9 E11.2-E11.9	
COPD in older adults	J41.0 J41.1 J41.8 J42 J43.0 J43.1 J43.2 J43.8 J43.9 J44.0 J44.1 J44.9 J47 J44.804	E84.0 E84.1 E84.8 E84.9 P25.0-P25.3 P25.8 P27.0 P27.1 P27.8 P27.9 Q25.4 Q31.1-Q31.9 Q32-Q34 Q39.0-Q39.4 Q89.3
Asthma	J45.0 J45.1 J45.8 J45.9 J46	E84.0 E84.1 E84.8 E84.9 P25.0-P25.3 P25.8 P27.0 P27.1 P27.8 P27.9 Q25.4 Q31.1-Q31.9 Q32-Q34 Q39.0-Q39.4 Q89.3
Hypertension	I10 I11.9 I12.9 I13.1 I13.9	
Heart failure	I11.0 I13.0 I13.2 I50.0 I50.1 I50.9	
Community-acquired pneumonia	J13 J14 J15.2-J15.4 J15.7 J15.9 J16 J16.8 J18.0 J18.1 J18.8 J18.9	D57.0-D57.3 D57.8 B20.0-B20.9 B59 C88.7 C88.9 C94.4 C94.5 D46.2 D47.0 D47.1 D47.9 D61.8 D70 D71 D72.0 D75.803 D76.1-D76.3 D80.0-D80.9 D81.0-D81.2 D81.4 D81.6-D81.9 D82-D84 D89.8 D89.9 E40-E43 I12.0 I13.1 I13.2 K91.2 N18.0 T86 Z94.0-94.4 Z94.8 Z99.2
Urinary tract infection	N10 N12 N15.1 N15.9 N16 N28.801 N28.820 N28.836 N30.0 N30.9 N39.0	N11 N13.0 N13.6 N13.7 N13.9 Q60.0-Q60.6 Q61.0-Q61.5 Q61.8 Q61.9 Q62.0-Q62.8 Q63 Q64.1-Q64.3 Q64.5-Q64.9 B20.0-B20.9 B59 C88.7 C88.9 C94.4 C94.5 D46.2 D47.0 D47.1 D47.9 D61.8 D70 D71 D72.0 D75.803 D76.1-D76.3 D80.0-D80.9 D81.0-81.2 D81.4 D81.6-D81.9 D82-D84 D89.8 D89.9 E40-E43 I12.0 I13.1 I13.2 K91.2 N18.0 T86 Z94.0-94.4 Z94.8 Z99.2

COPD, Chronic Obstructive Pulmonary Disease
