# Supplementary material for: Subgroups of High-Cost Patients and Their Preventable Inpatient Cost in Rural China
Source: Int J Health Policy Manag. 2024 Mar 9;13:8151. doi: 10.34172/ijhpm.2024.8151 (PMC11608279; doi:10.34172/ijhpm.2024.8151)
Supplement: Supplementary file 5 — The Healthcare Utilization and Spending for the 31 Clusters. [file ijhpm-13-8151-s005.pdf]

Article title: Subgroups of High-Cost Patients and Their Preventable Inpatient Cost in Rural China

Journal name: International Journal of Health Policy and Management (IJHPM)

Authors' information: Shan Lu[1,2,] Yan Zhang[1,2], Ting Ye[1,2]*, Dionne S. Kringos[3]

[1]School of Medicine and Health Management, Tongji Medical College, Huazhong University of Science and Technology, Wuhan, China.

[2]Research Centre for Rural Health Service, Key Research Institute of Humanities & Social Sciences of Hubei Provincial Department of Education, Wuhan, China.

[3]Amsterdam Public Health Research Institute, Department of Public and Occupational Health, University of Amsterdam, Amsterdam UMC, Amsterdam, The Netherlands.

***Correspondence to:** Ting Ye; Email: yeting@hust.edu.cn

Citation: Lu S, Zhang Y, Ye T, Kringos DS. Subgroups of high-cost patients and their preventable inpatient cost in rural China. Int J Health Policy Manag. 2024;13:8151. doi:10.34172/ijhpm.2024.8151

Supplementary file 5. The Healthcare Utilization and Spending for the 31 Clusters

Table S2 Utilization and spending for high-cost patient Clusters

Clusters	Utilization			Spending			
	Admissions (mean, SD)	Length of stay (mean, SD)	Outpatient visits (mean, SD)	Total spending, $ (mean, SD)	Preventable inpatient cost (%) [a]	Inpatient spending (%) [b]	OOP (%) [c]
Mixed chronic dis [d]	2.55(1.78)	9.6(8.71)	5.12(4.83)	3416.47(4472.06)	5.93	92.94	40.39
HF & COPD [e]	3.41(2.09)	9.39(3.28)	4.89(4.50)	3492.25(3203.87)	55.95	95.79	29.88
Hyperlipidemia & Hypertension	2.43(1.59)	8.79(5.27)	8.51(5.85)	3208.67(4836.8)	6.54	92.46	40.85
Cerebrovascular dis with headache	2.89(1.95)	9.2(6.01)	6.8(5.33)	3567.76(4501.17)	5.94	94.84	40.31
Rehabilitation of cerebrovascular	2.42(1.53)	20.35(26.63)	3.77(3.85)	8911.47(11567.36)	1.60	98.90	45.67
Chest pain [f]	2.84(1.92)	8.63(4.97)	6.2(5.22)	4194.07(5071.02)	7.87	95.64	40.57
CA & CHD [g]	2.52(1.53)	8.96(5.64)	5.64(5.97)	5454.26(5837.78)	6.87	95.83	47.70
Urinary calculi	2.45(1.60)	7.73(4.44)	4.45(4.40)	3081.15(3725.14)	6.15	96.69	41.98
Digestive [h]	2.13(1.45)	8.31(11.4)	3.84(3.87)	2704.78(4528.67)	3.31	96.05	44.80
Gallstone & cholecystitis	2.1(1.45)	8.36(3.92)	4.06(3.96)	2811.4(3561.51)	1.35	97.49	41.08
Disease of male reproductive system	2.5(1.79)	9.8(20.03)	5.68(5.62)	3211.82(2338.93)	6.08	96.57	38.65
Venous diseases which need surgery	2.22(1.95)	8.85(10.27)	4.85(5.50)	3506.42(3752.13)	2.91	97.26	42.58
Thyroid disorders	2.44(1.80)	8.28(4.72)	3.8(4.29)	3918.22(4974.78)	2.55	96.46	49.73

Hyperplasia [i]	2.76(2.39)	6.81(4.1)	3.95(4.99)	3483.52(4191.05)	1.49	96.05	48.22
Disease of Female reproductive system	2.43(1.72)	8.89(5.55)	3.96(3.84)	3325.63(4784.17)	1.47	96.50	44.37
Breast lesions	2.81(2.73)	6.03(4.06)	4.36(4.70)	3892.42(5166.68)	0.77	94.68	46.88
Digestive system tumour	4.55(3.24)	10.28(9.44)	4.25(4.37)	8793.02(8053.62)	0.71	97.42	46.79
Respiratory system tumour	4.69(3.3)0	10.78(8.95)	5.16(5.10)	9620.31(7991.76)	3.04	87.20	42.17
Cataract	3.34(1.67)	6.31(13.13)	4.95(4.97)	2559.47(2551.9)	9.92	94.30	34.06
Other eye disease	2.9(1.81)	6.95(4.15)	5.95(5.72)	3107.99(3769.46)	3.53	95.04	42.19
Respiratory infection	3.38(2.21)	9.01(9.10)	6.14(6.01)	4307.01(6670.16)	3.79	97.29	45.73
Child respiratory infection	3.5(1.87)	8.82(16.42)	2.74(3.96)	3134.79(7186.97)	0.17	97.32	51.64
Inflammatory dermatosis	3.08(2.26)	8.87(5.3)	7.45(5.45)	3063.53(3090.58)	9.49	95.36	40.81
Major skin disorders	2.46(1.94)	10.44(8.27)	8.26(7.74)	3435.23(6923.99)	4.59	95.22	45.96
Fracture	1.83(1.23)	12.51(20.40)	4.71(4.16)	3045.69(3309.71)	1.26	97.28	43.21
Liver disease	2.78(2.37)	9.43(10.44)	5.21(4.84)	6113.13(8163.91)	2.13	89.43	45.32
VFC [j]	2.77(2.20)	8.76(6.27)	7.34(6.02)	3653.7(5927.57)	4.78	92.00	43.31
Mental disease	2.84(2.38)	32.11(28.78)	4.85(4.17)	3707.47(2788.42)	2.78	91.98	32.45
Arthritis	2.9(2.01)	9.03(5.67)	4.92(5.29)	3305.3(4283.63)	6.06	91.12	44.63
Renal failure	12.21(9.26)	13.05(8.52)	4.45(4.91)	13586.94(10072.84)	1.66	92.79	23.50
Other neurological disorder	3.28(2.45)	11(10.98)	5.03(5.03)	4615.74(6408.62)	8.82	92.94	41.77

a. Proportion of preventable inpatient cost on total spending.

b. Proportion of inpatient spending on total spending.

c. Proportion of out-of-pocket (OOP) spending on total spending.

d. Mixed chronic disease.

e. Heart failure and chronic obstructive pulmonary disease.

f. Circulatory system disorders with chest pain.

g. Cardiac arrhythmias and coronary heart disease.

h. Digestive system diseases which need surgery (Such as appendicitis, haemorrhoids, polyps, and hernias).

i. Non malignant hyperplasia of head, neck, ear, nose, pharynx or mouth.

j. Vertigo syndrome and fever and cerebral infarction.
